# A Program for the Study and Support of Caregivers: An Innovative Model for US Medical Schools

**DOI:** 10.1093/geroni/igaf122.177

**Published:** 2025-12-31

**Authors:** Sara Czaja, Mark Lachs

## Abstract

Formal (paid) and informal (unpaid) caregivers play crucial roles in supporting the health and well-being of older adults with chronic and disabling health conditions. The physical and emotional demands of caregiving are profound, yet caregivers are not systematically engaged or supported in health care settings. The Program for the Study and Support of Caregivers at Weill Cornell Medicine (WCM) is the first program of its kind within a US medical school. It convenes a multidisciplinary team of scientists, clinicians, and educators to address the most pressing topics and knowledge gaps in the field of caregiving. A unique feature of our Program is the synergistic integration between our research patient care, and educational programs, which allows us to implement and disseminate findings to our patient and caregiving populations. This symposium is organized according to the Program’s three pillars: discover, care, teach. Following the first presentation, which will provide an overview of the Program’s vision, structure, and unique features (Czaja), subsequent presentations will describe (1) our world class research component that aims to address pressing gaps in the research landscape by designing, adapting, and implementing evidence-based programs (Sterling), (2) innovative practice-based initiatives that will promote caregiver assessment, education, and support in clinical practice (Adelman), and (3) comprehensive educational programing utilizing didactic teaching, reflective practice, and experiential learning methods to prepare trainees to deliver high-quality, family-centered care (Phongtankuel).Our discussant Dr. Lachs, will lead a conversation about the Program’s significance and potential to serve as model for the study and support of caregivers nationwide.

